# Sequences Located within the N-Terminus of the PD-Linked LRRK2 Lead to Increased Aggregation and Attenuation of 6-Hydroxydopamine-Induced Cell Death

**DOI:** 10.1371/journal.pone.0045149

**Published:** 2012-09-13

**Authors:** Neeraj Pandey, Mark T. Fahey, Yuh-Jiin I. Jong, Karen L. O'Malley

**Affiliations:** Department of Anatomy and Neurobiology, Washington University School of Medicine, St. Louis, Missouri, United States of America; National Institutes of Health, United States of America

## Abstract

Clinical symptoms of Parkinson's disease (PD) arise from the loss of substantia nigra neurons resulting in bradykinesia, rigidity, and tremor. Intracellular protein aggregates are a pathological hallmark of PD, but whether aggregates contribute to disease progression or represent a protective mechanism remains unknown. Mutations in the leucine-rich repeat kinase 2 (LRRK2) gene have been linked to PD in both familial cases and idiopathic cases and aggregates of the LRRK2 protein are present in postmortem PD brain samples. To determine whether LRRK2 contains a region of protein responsible for self-aggregation, two independent, bioinformatic algorithms were used to identify an N-terminal amino acid sequence as being aggregation-prone. Cells subsequently transfected with a construct containing this domain were found to have significantly increased protein aggregation compared to wild type protein or a construct containing only the last half of the molecule. Finally, in support of the hypothesis that aggregates represent a self-protection strategy, aggregated N-terminal LRRK2 constructs significantly attenuated cell death induced by the PD-mimetic, 6-hydroxydopamine (6-OHDA).

## Introduction

Parkinson’s disease (PD) is a common neurodegenerative disorder with pathological hallmarks that include the loss of dopaminergic substantia nigra neurons and the development of intracytoplasmic protein aggregates termed Lewy bodies [1]. Although most PD cases are idiopathic, a number of PD-linked genes have been discovered [2], [3]. Of these, mutations in *LRRK2* (Leucine-rich repeat kinase 2) are the most common genetic cause of PD, found in about 5% of familial cases and 1-2% of idiopathic cases [4]. LRRK2 codes for a large 2527-residue protein that contains a leucine-rich repeat region, a Ras of Complex Proteins (ROC) GTPase domain, a C-terminal of ROC (COR) domain, a kinase domain with homology to the mixed lineage kinases (MLK), and a WD-40 interaction domain at the C-terminus [4], [5]. In PD patients mutant forms of LRRK2 protein have been associated with Lewy bodies [4] as well as with tau pathology [6], ubiquitin and/or TDP-43 positive inclusion bodies [7] although whether LRRK2 is playing a dominant role is still controversial [8]. Abnormal granular LRRK2 staining has also been found in the substantia nigra where it appears to co-localize with endosomal or lysosomal markers [9]. Caution is warranted, however, since even in populations with the same mutation variable pathology is observed [10].

When expressed in cultured cells or primary neurons, mutant forms of LRRK2 also form cytoplasmic inclusion bodies or aggregates [11]. For example, the pathogenic mutations R1441C, Y1699C, and I2020T form pronounced aggregates in a large proportion of cultured cell types [11], [12], [13]. Although earlier studies indicated that overexpression of the prevalent G2019S mutation also resulted in a large percentage of aggregates [11], a recent report examining 41 different LRRK2 mutations showed only diffuse cytoplasmic staining [13]. Intriguingly, this same study found that sequences upstream of the LRR domain of LRRK2 bound 14-3-3 proteins in a phosphorylation-dependent fashion; cells expressing mutations that disrupted this interaction developed aggregates in their cytoplasm [13]. These data suggest that sequences in the first half of the protein may exert control over the latter half of the molecule. In support of this notion, expression studies exploring differences between LRRK2 and LRRK1 noted that LRRK2 with its unique N-terminus produced aggregates whereas the closely related LRRK1, did not [12]. Taken together these data suggest that there might be a domain or region within the LRRK2 N-terminus that is responsible for aggregation or that masks a domain such as the 14-3-3 binding site that normally would prevent this process.

Structurally, the N-terminal domain of LRRK2 is composed of 14 so-called LRRK2-specific repeats (amino acids [aa] 20–680) [14] followed by seven ankyrin-like repeats (aa 690–860) [15]. Both of these structures are thought to be protein-interaction domains. Recently, Lu et al., [16] succeeded in purifying the N-terminal domain of LRRK2. Using Mass Spectroscopy and circular dichroism, Lu et al., [16] showed that the N-terminus of LRRK2 exists as an oligomer in solution which might reflect a propensity for protein aggregation [16]. Although the exact function of the N-terminal region of LRRK2 is unknown, several new N-terminal coding sequence variants have been discovered in large populations and different ethnic groups [17].

Using bioinformatics techniques, LRRK2 deletion constructs, high-content imaging, and western blots, the present study identifies a highly aggregation-prone region in the previously unstudied N-terminus of the LRRK2 protein. Interestingly, the aggregation-prone N-terminal LRRK2 conferred a protective effect on cells when challenged with the PD neurotoxin, 6-OHDA.

## Materials and Methods

### Bioinformatic identification of LRRK2 aggregation-prone domains

The PASTA (prediction of amyloid structure aggregation) algorithm by Trovato et al. [18], [19] is publicly accessible at http://protein.cribi.unipd.it/pasta/. The Tartaglia et al. [20], [21] algorithm was applied to full-length WT LRRK2 in window sizes from 5–30 amino acids and the five most aggregation-prone segments were retained, pooled, and plotted using a Linux shell script generously provided by François Marchand and Amedeo Caflisch , University of Zurich, Zurich Switzerland.

### Creation of LRRK2 clones and constructs

Wild type LRRK2^1–2527^ (WT-LRRK2), N-terminal LRRK2^1–938^ (N-term-LRRK2), and C-terminal LRRK2^926–2527^ (C-term-LRRK2) were created by restriction digestion and PCR in a zeocin and ampicillin-resistant pcDNA 3.1 plasmid backbone. Thirty-five computationally-determined aggregation-prone amino acids (269–303) were deleted from the N-terminal construct to produce N-term deleted LRRK2^Δ1–938^ (N-del LRRK2). All constructs were tagged at the N-terminus with Enhanced Green Fluorescent Protein (EGFP). The WT, N-terminal, and N-terminal deleted constructs were also tagged with an HA motif at the N-terminus. Constructs were confirmed by DNA sequencing. HA-only WT and N-terminal constructs were also created by deletion of the EGFP tag from the N-term-LRRK2.

### Cell cultures

Human neuroblastoma SH-SY5Y cells (obtained from Dr. Dawson TM, The Johns Hopkins University School of Medicine, Baltimore, MA, [22] were grown in DMEM containing 10% fetal bovine serum, and 1x minimal essential medium non-essential amino acids at 37°C. For primary cultures, the ventral mesencephalon was removed from embryonic day 14 (E14) CF1 murine embryos (Charles River Laboratories, Wilmington, MA). Tissues were mechanically dissociated, incubated, and plated as described in [23].

### Transfection and quantitation of EGFP-LRRK2 aggregation in SH-SY5Y cells

SH-SY5Y cells were plated at 50,000/well in Poly-D-Lysine (PDL)-coated 24 well plates and transiently transfected with 2 µg of EGFP-LRRK2 (WT-, N-term-, C-term-, or N-del-) plasmid DNA using Fugene HD (Roche, Nutley, New Jersey). The relative aggregation propensities of the WT, C-term, N-term, and N-term-deleted LRRK2 constructs were measured using an automated high-content ImageXpress Micro imaging system (Washington University School of Medicine Chemical Genetics Screening Core). At each time point, twenty-five 40X images were acquired from sites evenly distributed across each well. Four wells per condition were analyzed such that 100 images/sample were assessed. Data are expressed as means ± S.E.M. of at least 3 independent experiments. Images were automatically analyzed using the ImageXpress Granularity Function which calculated average number of granules per site, total area of granules per site, and average intensity of granules per site. An aggregate was defined as anything between 1–12 micrometers and 2000 grey levels over the local background.

### Transfection and quantitation of HA-LRRK2 aggregation in SH-SY5Y cells

SH-SY5Y cells were plated in PDL-coated P35 dishes (300,000/dish) and transfected with 2 µg of HA-WT-LRRK2 or HA-N-term-LRRK2 using 4 µl of Lipofectamine LTX (Invitrogen). Cells were fixed after 16 hours and immunostained with monoclonal anti-HA (1∶400, Babco, Richmond, CA). Twenty-four random fields of HA-tag labeled cells were quantitated using a 40X objective by counting HA-expressing and HA-expressing cells with aggregates in each. Data are expressed as means ± S.E.M. of 3 independent experiments with multiple dishes and a total of about 2000 cells analyzed. Cells were also plated on PDL-coated 12 mm diameter glass coverslips (50,000/coverslip) and co-transfected with LRRK2 construct (HA-WT-LRRK2 or HA-N-term-LRRK2) in a 15∶1 mass ratio (1.5 µg: 0.1 µg) with 4 µl of Lipofectamine LTX. Cells were fixed after 16 hours and immunostained with monoclonal anti-HA (1∶400). Data are expressed as means ± S.E.M. of independent experiments with multiple coverslips and a total of about 500 HA-labeled aggregates analyzed.

### Biochemical analysis of LRRK2 constructs in SH-SY5Y cells

EGFP-LRRK2 constructs (WT-, N-term-, C-term-) as well as HA-LRRK2 (WT, N-term) were transfected in SH-SY5Y cells as described above and then harvested 16 h later. Lysates were fractionated based on their differential solubility using buffered solutions as described procedure [24]. Cells were washed three times in ice-cold PBS, and samples were harvested in 25 mM Tris-HCl, pH 7.5, 150 mM NaCl, 1 mM EDTA, 1% Triton X-100, and a mixture containing 1 mM phenylmethylsulfonyl, complete protease inhibitor cocktail (Roche, Indianapolis, MO) and phosphatase inhibitor II cocktail (Sigma-Aldrich, St. Louis, MO). Samples were sedimented at 100,000× *g* for 30 min at 4°C. Supernatants were removed, and pellets were sonicated in 1.5× Laemmli sample buffer. SDS sample buffer was added, and samples were heated to 100°C for 5 min before Western blot analysis. Equal proportions of supernatant (Triton-soluble protein) and pellet (Triton-insoluble protein) were loaded and analyzed as the percentage pelleted (Image J software; NIH).

### Transfection and quantitation of aggregates in primary mid-brain cultures

Div 6 (6 days *in vitro*) primary mid-brain cultures were transfected with 2 µg EGFP-LRRK2 (WT-, N-term-, C-term, or N-del-) plasmid DNA using 4 µl of Lipofectamine 2000 (Invitrogen). After 24 hours, cultures were fixed and immunostained with monoclonal anti-MAP2 (microtubule-associated protein 2, 1∶500, Millipore, Billerica, MA) or polyclonal anti-TH (tyrosine hydroxylase, 1∶1000, Pel-Freez, Rogers, AK) antibody. MAP2 or TH positive neurons with EGFP-expressing aggregates were quantitated using a 40X objective. Data are expressed as means ± S.E.M. of 3 independent experiments with multiple dishes and a total of about 600 MAP2 or 150 TH positive neurons analyzed.

### Immunocytochemistry

Cells were fixed, blocked, and probed with primary and secondary antibodies as described [23]. Secondary antibodies included goat anti-rabbit and/or anti-mouse Cy3 (both at 1∶500; Jackson ImmunoResearch, West Grove, PA) and goat anti-mouse Alexa 488 (1∶300; Molecular Probes, Eugene, OR).

### Western Blots

EGFP-LRRK2 (WT-, N-term-, C-term-, or N-del-) transfected SH-SY5Y cells were lysed and processed for western blotting as described [25]. Protein concentrations were determined by Bradford assay (Biorad, Richmond, CA) using a BSA (bovine serum albumin, New England Biolabs, Ipswich, MA) protein standard curve. Proteins were separated by SDS-PAGE, blotted, and probed with monoclonal anti-HA (1∶1000) or polyclonal anti-LRRK2 (1∶1000, C-terminal residues 2500–2527, Novus Biologicals). A horseradish peroxidase conjugated with goat anti-rabbit IgG (1∶2000, Cell Signaling Technology, Inc., Beverly, MA) or anti-mouse IgG (1∶2500, Sigma-Aldrich) was used in conjunction with enhanced chemiluminescence (GE Healthcare, Piscataway, NJ) to detect the signal.

### Determination of cell viability

SH-SY5Y cells (50,000 cells/well in 24-well plates) were transfected with 0.5 µg EGFP-LRRK2 (WT, N-term, C-term, or N-del) plasmid using LipoD293 (SignaGen Laboratories, Ijamsville, MD) to achieve more than 90% transfection efficiency. Three days after transfection, cultures were stained with propidium iodide (PI, 5 µg/ml) for 5 minutes to detect cell death. Alternatively, cultures were treated with 100 µM 6-OHDA (Sigma-Aldrich) 24 hours after transfection. Cell survival assays were performed after 24 hours using MTT (3-(4,5-dimethylthiazol-2-yl)-2,5-diphenyltetrazolium bromide) as described [26]. The number of living cells for each construct was expressed as the percentage absorbance of the 6-OHDA-treated samples to the construct-specific control.

### Statistics

Data were analyzed by Student's *t* test or one-way ANOVA with Bonferroni *post hoc* test for multiple comparisons. The mean ± SEM represents the average of the mean value from each of the experiments. Summary of statistical data are shown in Tables S1, S2, S3, S4, S5, S6, S7).

## Results

### Aggregation algorithms predict a novel aggregation-prone domain in the N-terminus

**Figure 1:**
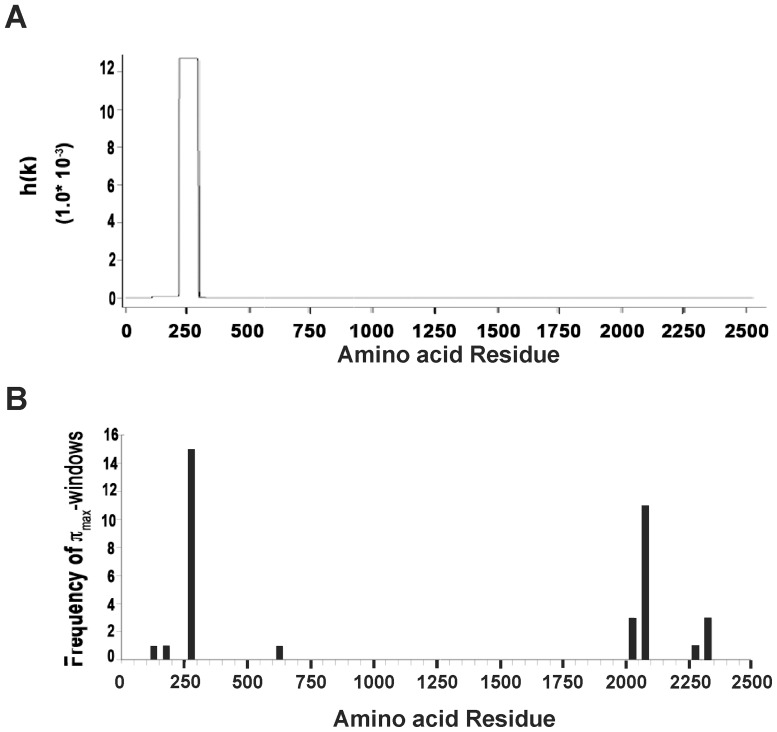
Predicted LRRK2 aggregation domains. (**A**) The PASTA algorithm by Trovato [19] predicts an aggregation domain at 210–310 amino acid residues in the LRRK2 protein (NCBI accession number NP_940980). (**B**) An independent aggregation algorithm [21] predicts two aggregation domains, one peak between amino acid residues (250 to 299) overlapping with the N-terminus region predicted by PASTA (A) and the other at amino acid residues 2050–2099. Tick marks in B represent sliding window of 50 amino acids.

Based on the recent finding that the N-terminus of LRRK2 could form oligomers [16] and other studies suggesting that this region might be involved in aggregation, we used two independent aggregation domain prediction methods to determine if such a domain (s) existed in the LRRK2 sequence. Interestingly, the so-called PASTA algorithm predicted an aggregation-prone region at 210–310 amino acids of the LRRK2 protein (Fig. 1A) [19]. Sequences encompassing this region (amino acid residues 250–299; Fig. 1B) were also predicted to serve as an aggregation domain by a second algorithm, that of Tartaglia et al., [21]. The latter algorithm also predicted a second aggregation domain at 2050–2099 of the LRRK2 protein (Fig. 1B).

### N-terminus of LRRK2 shows increased aggregation

**Figure 2:**
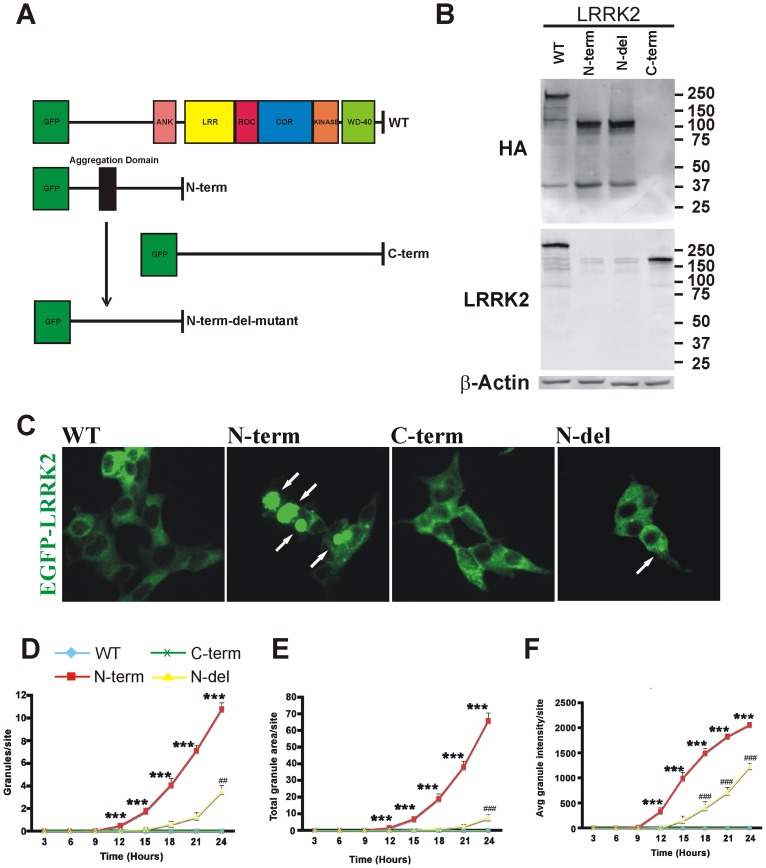
LRRK2 N-terminal sequences show increased aggregation. (**A**) Schematic of WT, N-term, and C-term showing locations of functional groups, EGFP tag, and bioinformatically-determined N-terminal aggregation-prone region. N-terminal deleted LRRK2 was created by the removal of 35 amino acids including the aggregation region. (**B**) Western blots of cell lysates of SH-SY5Y cells transfected with indicated LRRK2 construct showed equal expression of the LRRK2 protein with anti-HA (top panel) or anti-C-terminal- (bottom panel) LRRK2 antibody. Equal lysate loading is indicated by β-actin loading control. (**C**) EGFP-LRRK2 (WT, N-term, C-term, or N-del) constructs were transfected into SH-SY5Y cells. Representative images after 24 hours show N-term-LRRK2 cells with increased aggregates as indicated by arrows. Quantitation of granule number (**D**), granule size (**E**), and granule intensity (**F**) over 24 hour time course using ImageXpress automated acquisition and analysis system. One hundred images were analyzed per construct across 4 wells. Line graphs show the average of the mean values from each of three experiments ± S.E.M., (*** denotes p value <0.001 for N-term versus WT, C-term or N-del- LRRK2 construct. ^###^ denotes p value <0.001, ^##^ denotes p value <0.01 for N-del versus WT, C-term or N-term-LRRK2 construct, n = 4, one-way ANOVA with Bonferroni *post hoc* test).

To test whether the N-terminus of LRRK2 did indeed contain an aggregation domain, constructs were generated of EGFP-fused wild type LRRK2 (1–2527 amino acids, WT-LRRK2), N-terminal LRRK2 (1–938 amino acids, N-term LRRK2), C-terminal LRRK2 (926–2527, C-term- LRRK2), and N-terminal LRRK2 with a deletion of 35 amino acids (residues 269–303, N-del-LRRK2) encompassing the computationally-identified aggregation-prone region. The N-terminal and WT LRRK2 clones also contained an HA tag immediately downstream of EGFP (Fig. 2A). Following transient transfection of neuroblastoma SH-SY5Y cells, western blot analysis of cell lysates showed bands at the predicted molecular weight of each construct using anti-HA, anti-C-terminal-LRRK2 (Fig. 2B) or anti-GFP antibody (not shown). The blots also showed equal expression of each LRRK-2 protein in transfected cells (Fig. 2B). In order to compare and quantitate results from each construct, SH-SY5Y cells were transfected and imaged every three hours for 24 hours using the ImageXpress high-content, automated microscopy system to avoid bias. Signs of aggregation were observed in cells transfected with N-term-LRRK2 as early as 12 hours post transfection whereas cells expressing WT-LRRK2 or C-term-LRRK2 exhibited no aggregates over this time period (Fig. 2C). The number, size and intensity of N-term-LRRK2 granules demonstrated rapid growth with time and were highly significant (Fig. 2D-F, Tables S1, S2, S3). In contrast, deletion of the identified 35 amino acid “aggregation” region in LRRK2^Δ1–938^ (N-del-LRRK2) resulted in significantly reduced granule size, intensity and number at all time points measured (Fig. 2C – F, Tables S1, S2, S3). By three days after transfection, WT- and C-term LRRK2 did show formation of granules. However, the number, size and intensity of these granules were significantly lower than those detected with N-term-LRRK2 (data not shown). Thus at least some aspects of the aggregation process still occurred with WT-, C-term and N-del-LRRK2 albeit slower. These data are consistent with the notion that the N-terminus of LRRK2 contains a region between amino acids 269 and 303 that promotes aggregation.

### Increased aggregation in HA-N-term-LRRK2 in SH-SY5Y cells

**Figure 3:**
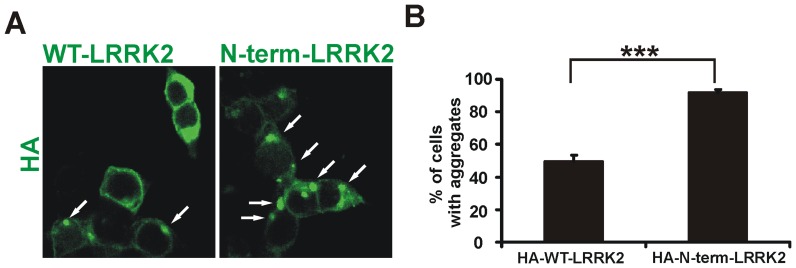
HA-tagged LRRK2 constructs show same pattern of aggregation. (**A**) SH-SY5Y cells were transfected with LRKK2 constructs and immunostained with anti-HA antibody after 16 hours. Arrows point to intracellular aggregates. (**B**) Quantitation of HA-LRRK2 cells with aggregates; Ninety-two percent of N-term-HA-LRRK2 cells showed aggregates compared to 49% of HA-WT-LRRK2 cells. Bars represent the average of the mean values from each of three experiments ± S.E.M. (***P<0.001, n = 3, Student's *t*-test).

To exclude the possibility that the EGFP tag itself could influence the aggregation of LRRK2 proteins, we repeated these experiments using HA-tag –LRRK2 constructs. HA-WT-LRRK2 or HA-N-term-LRRK2 constructs were transfected into SH-SH5Y cells and the percentage of HA-labeled cells with aggregates was measured as described. Increased aggregation formation (91.55±2.24%) was observed in HA-N-term-LRRK2 compared to HA-WT-LRRK2 (49.43±3.72%) expressing cells (Fig. 3A, B, Table S4) consistent with the observation that the N-terminus contains sequences that induce aggregation.

### N-term-LRRK2 is more insoluble than WT-LRRK2 in SH-SY5Y cells

**Figure 4:**
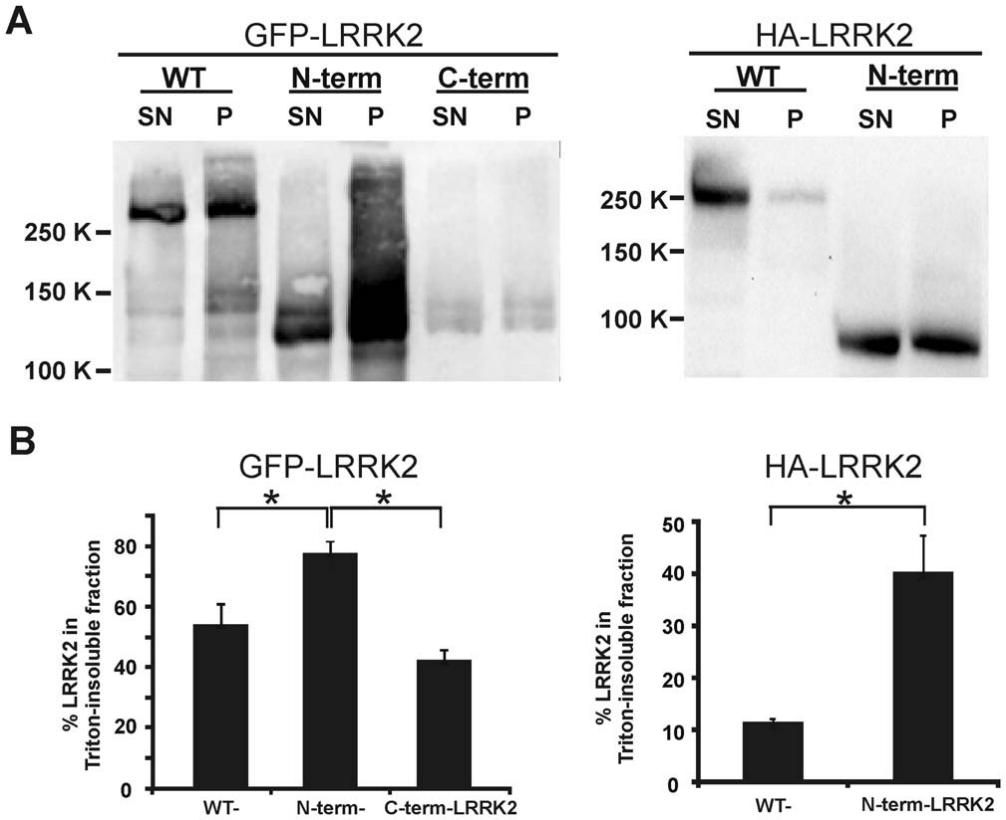
N-term-LRRK2 is more insoluble than WT-LRRK2 in SH-SY5Y cells. SH-SY5Y cells were transfected with EGFP-LRRK2 (WT, N-term, C-term) or HA-LRRK2 (WT, N-term) constructs and harvested 16 h later. Cells were lysed and fractionated as described in the Methods followed by western blot analysis. (A) Representative immunoblots showing LRRK2 bands with anti-HA antibody, SN (supernatant, Triton-soluble fraction), P (pellet, Triton-insoluble fraction). (B) Quantitation of multiple experiments in which the percentage of Triton-insoluble LRRK2 was calculated as Triton-insoluble LRRK2/(Triton-soluble + Triton-insoluble LRRK2) * 100. Data represent the mean ± SD (*p<0.05, n = 3).

To further characterize the biochemical nature of the N-term-LRRK2 aggregates, cells transfected with the different LRRK2 constructs were fractionated to separate the soluble cytosolic portion (supernatant) from the insoluble aggregated form (pellet) of LRRK2. Western blots of fractionated lysates showed that EGFP-N-term-LRRK2 contained about 78.2%±4.3% pelleted LRRK2 whereas EGFP-WT-LRRK2 cells and C-term-LRRK2 contained 54.3%±6.5% and 42.8%±3.3% insoluble protein respectively (Fig. 4). Even larger increases of Triton insoluble N-term-LRRK2 (40.3%±7.0%) compared to WT-LRRK2 (11.5%±0.6%) were detected in cells transfected with HA-LRRK2 constructs (Fig. 4B, Table S5). These data support the notion that the LRRK2 N-terminus contains sequences that induce aggregation.

### Increased N-term-LRRK2 aggregation in primary midbrain neurons

**Figure 5:**
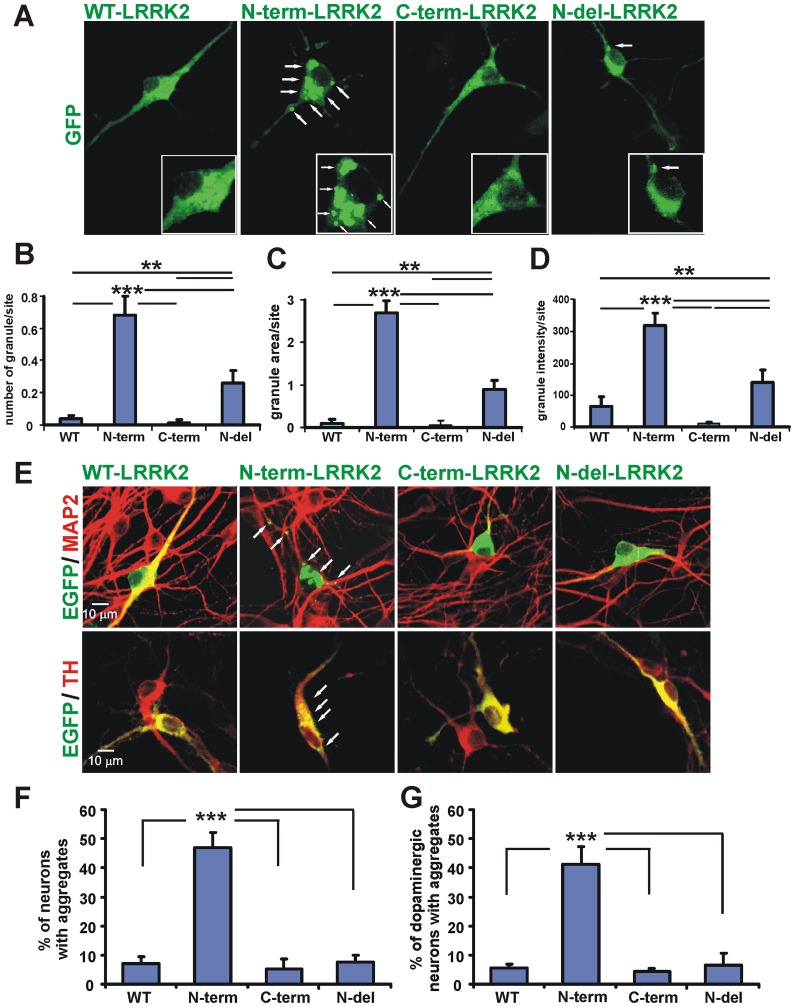
Increased N-term-LRRK2 aggregation in mesencephalic neurons. (**A**) Representative images showing N-term-LRRK2 cells with increased high intensity aggregates (arrows) in the cell body and neurites compared to WT-, C-term-, or N-del-LRRK2 constructs. Inset of the cell body is enlarged (bottom) to illustrate aggregates (arrows). Aggregates were quantitated utilizing number of granules/site (**B**), total granule area/site (**C**), and average granule intensity per site (**D**). Data shown are average of 3 independent experiments with more than 6 dishes/construct and 64 images/dish. (** P<0.01, ***P<0.001, one-way ANOVA with Bonferroni *post hoc* test). (**E**) Representative images showing the colocalization of EGFP-LRRK2 with MAP2 or TH immunostaining. Increased aggregates were observed in N-term-LRRK2 neurons including dopaminergic neurons. (**F**) Quantitation of numbers of LRRK2/MAP2 positive neurons with aggregates. Bars represent means of three experiments ± S.E.M. with multiple dishes and a total of about 600 MAP2 positive neurons analyzed. (*** P<0.001, one-way ANOVA with Bonferroni *post hoc* test). (**G**) Quantitation of numbers of LRRK2/TH-positives neurons with aggregates. Bars represent mean values from each of 4 experiments ± S.E.M. A total of about 150 dopaminergic neurons were analyzed (*** P<0.001, one-way ANOVA with Bonferroni *post hoc* test).

To test whether aggregation occurs in primary neurons, mouse midbrain cultures were transfected with EGFP-LRRK2 (WT, N-term, C-term, or N-del) and aggregates were measured after 24 hours. Significantly increased numbers of granules, total granule area and average granule intensity were detected in the cell body and neurites in N-term-LRRK2 cells while WT, C-term and N-del LRRK2 had fewer and smaller aggregates (Fig. 5A–D). Granules were visualized within neurites at least 30 µm away from the soma in N-term-LRRK2 transfected neurons. Removal of the aggregation–prone region from the N-terminus significantly reduced LRRK2 aggregation (Fig. 5A–D, Table S6). N-term-LRRK2 aggregation is neuron-specific since all of the EGFP-LRRK2 expressing cells co-localized with MAP2 (Fig. 5E, top panel). More than 47% of the N-term-LRRK2/MAP2 cells possessed aggregates while less than 8% of the WT (7.02±1.7%), C-term (5.33±2.36%) or N-del (7.31±1.64%) LRRK2/MAP2 cells did (Fig. 5F, Table S6). Finally, N-term-LRRK2 aggregation also occurred in identified dopaminergic neurons showing increased aggregation (43.3±3.04%) compared to less than 6% of WT (4.89±0.64%), C-term (4.25±0.25%), or N-del (5.22±0.95%) -LRRK2 in neurons co-stained for the biosynthetic enzyme, tyrosine hydroxylase (TH; Fig. 5E bottom panel, G).

### N-term-LRRK2 attenuates 6-OHDA induced cell death in SH-SY5Y cells

**Figure 6:**
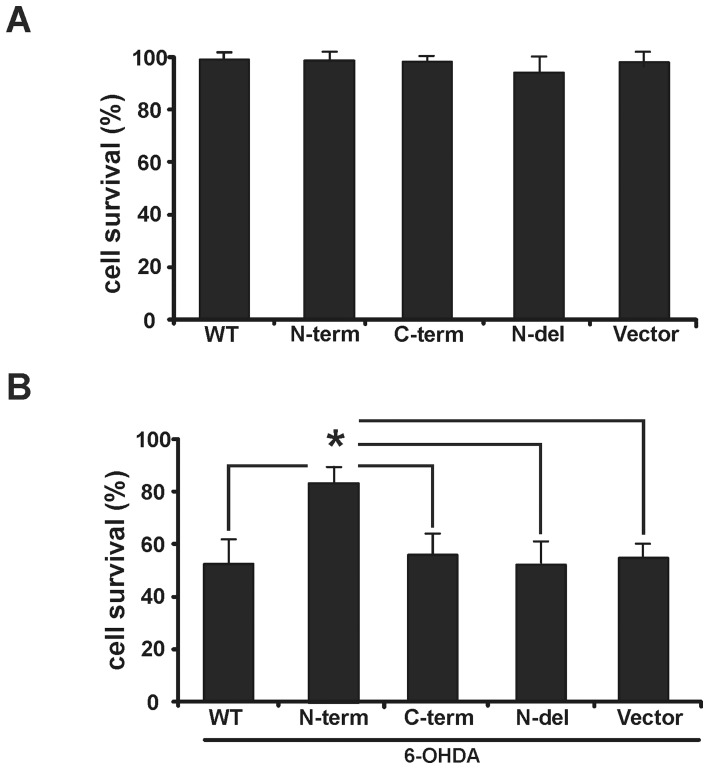
N-term-LRRK2 attenuates 6-OHDA-induced cell death. (**A**) Indicated LRRK2 construct was transfected into SH-SY5Y cells and the cell viability was assessed using PI staining after 72 hours. No significant cell death was detected for any construct. Bars represent mean values from each of three experiments ± S.E.M. (**B**) Cells were treated with 100 µM 6-OHDA 24 hours after transfection. Cell viability after 24 hours was assessed using the MTT reduction assay. Bars represent % survival versus untreated control. Bars represent mean values from each of 4 experiments ± S.E.M. (*P<0.05, n = 4, one-way ANOVA with Bonferroni *post hoc* test).

To determine whether aggregation led to a loss of cell viability, EGFP-LRRK2 constructs (WT, N-term, C-term, or N-del) were transfected into SH-SY5Y cells as described. Cells were followed for up to 72 hours with no evidence of cell death as ascertained by morphology, trypan blue exclusion, activated caspase 3 staining, and or changes in propidium iodide staining (Fig. 6A and not shown). Thus even cells expressing massive N-term-LRRK2 aggregates did not exhibit increased toxicity. In order to determine whether aggregation might serve a protective role, we challenged cells expressing the various constructs with the PD-mimetic, 6-OHDA. SH-SY5Y cells expressing WT, C-Term, and N-term-del LRRK2 constructs all exhibited the same percentage of cell survival as did vector-only cells in the presence of this toxin (Fig. 6B, Table S7). In contrast the N-term-LRRK2 exhibited significantly increased cell viability (Fib. 6B, Table S7). No basal effects on MTT reduction were observed amongst the different constructs. Although there may be other explanations, under these conditions the formation of aggregates would appear to be protective in the presence of a free-radical generating toxin.

## Discussion

LRRK2 mutations exhibit a variety of pathological characteristics which include α-synuclein, ubiquitin, and tau protein inclusions leading to the suggestion that LRRK2 may be an important upstream modulator of protein aggregation in neurodegenerative disease [1], [4], [5], [11]. Studies have also shown that LRRK2 itself can form aggregates both in vivo and in vitro [1], [8], [10], [11], [24], [27]. Using two different unbiased protein aggregation algorithms combined with high throughput image content analysis, the present study shows that an aggregation-prone domain exists in the N-terminus of LRRK2. Cultured cells or primary neurons transfected with this domain exhibit higher levels of aggregated material whereas when this region is deleted significantly fewer and smaller aggregates are formed. Finally, aggregated N-terminal LRRK2 significantly attenuated cell death induced by the PD-mimetic, 6-OHDA. Therefore, sequences within the N-terminus of LRRK2 may affect its cellular distribution and interaction with other proteins both in terms of normal functions as well as those disrupted in PD.

In the last few years several computational models have been developed to predict the propensity of a given sequence to form extended β-conformations resulting in amyloid-like fibrils [28]. One such algorithm, prediction of amyloid structure aggregation (PASTA), is based on the orientation of neighboring β-sheet strands to adopt minimal energy conformations for improved stability [18], [19]. In contrast, the computational model developed by Tartaglia and coworkers [21] uses not only the physicochemical properties of β-aggregation but also a more empirical approach of breaking sequences into overlapping segments which can be compared to known amyloidogenic peptides. Importantly both approaches have yielded comparable results emphasizing the validity of such strategies in defining aggregation prone “hot-spots”. The specific sequences identified (210–310 and 250–299 amino acid residues respectively) encompass the LRRK2-specific repeats 5–7 [14]. In particular the sequence FFNILVLN between LRRK2 N-terminal repeats 6 and 7 is almost completely conserved throughout evolution as far back as echinoderms and cnidarians [14]. Deletion of this region decreased the ability of the LRRK2 N-terminal fragment to form granules in terms of number, size and intensity (Fig. 2, 3, 5) as well as its propensity to enhance cell survival (Fig. 6). Data base searches using the 35 amino acids that were deleted in N-term-del (269–303) did not reveal any significant homologies with other proteins known to aggregate suggesting that this sequence is unique. Taken together, these findings implicate a region in the N-terminal domain of LRRK2 as being involved in LRRK2's cellular distribution.

In many disorders involving protein aggregation, it is the smaller fibrils/oligomers that are toxic; larger aggregates are thought to represent cell protective mechanisms [29], [30]. Processes such as protein folding and protein clearance have been widely implicated in neurodegeneration and not uncommonly unfolded protein response is triggered when cellular systems are overwhelmed. Previously we have shown that 6-OHDA triggers the unfolded protein response in cell lines and primary dopaminergic neurons [25], [31], [32]. Despite the extremely large aggregate formation (Fig. 2, 3, 5), however, none of the LRRK2 constructs triggered this response, at least in terms of increased Bip, CHOP, IRE1 expression or XBP-1 cleavage (data not shown).

### N-term-LRRK2 aggregates are cell protective

The role of protein aggregation in neurodegenerative disease is still unclear. Aggregates may contribute to the pathological process, be a correlating sign of disease, or act as a cell protection mechanism. Our study supports the protective hypothesis as 6-OHDA-induced cell death was significantly attenuated in the N-term-LRRK2 transfected SH-SY5Y cells (Fig. 6B). This effect was absent both in the vector control and in N-del-LRRK2 transfected cells, supporting the notion that the N-term-LRRK2-mediated high levels of aggregation were responsible versus the absence of the C-term-LRRK2 (Fig. 6B). Likewise, aggregate formation in Huntington's disease has been shown to prolong cell life relative to cells without inclusions [33]. Although this effect has been attributed to the sequestering of toxic fibril intermediaries, huntingtin aggregates have also been shown to induce autophagy and thereby reduce toxicity [34]. Autophagy induction can protect against apoptosis by speeding the clearance of damaged mitochondria as well as reducing levels of cytochrome c and activated caspase to promote cell survival [35]. Consistent with these data are previous reports showing that 6-OHDA treatment rapidly induces mitochondrial fission in SH-SY5Y cells [36] and that 6-OHDA toxicity is reduced in transgenic mice over-expressing mitochondrial MnSOD versus control mice [37]. Conceivably, N-term-LRRK2 induction of autophagy might help clear 6-OHDA-induced mitochondrial stress and fragmentation. Surprisingly, despite the very large aggregates seen in N-term-LRRK2 transfectants, no sign of unfolded protein response was observed (data not shown). Given that 6-OHDA induces unfolded protein response in dopaminergic cell lines and primary dopamine neurons [31], [32], it would be of interest to determine whether the N-term-LRRK2 transfectant blocked toxin-challenged cells from mounting a stress response.

What physiological role does the N-terminal domain of LRRK2 play? A recent systematic study of LRRK2 exonic variants revealed several rare variants with differences in frequency between PD patients and controls within the domain of interest (A211V, C228S, N238I) [17]. Although large meta-analyses will be required before the roles of these rare variants are fully described, the new associations are consistent with the possibility that the N-terminal region might contribute to the development of PD. To further understand the role of this domain, it will be important to characterize the intramolecular regulation of LRRK2 and to show how loss of the N-terminus might regulate kinase activity, affect interactions between the different domains, affect dimerization, toxicity, intracellular distribution, the scaffolding properties of the protein and whether these manipulations lead to additional pathogenic effects. In theory any such variant might alter protein function and thus contribute to a person's susceptibility towards PD. Besides PD, LRRK2 has also been associated with susceptibility to Crohn's disease and leprosy [38]. Thus, the N-terminal region defined here might be important for protein/protein interactions in systems other than neurons. At present our study does not address these possibilities.

Others have reported that LRRK2 produces aggregates in cell culture systems whereas the closely related LRRK1, does not [12] suggesting a role for N-terminal LRRK2-specific repeats in aggregation. That sequences in the first half of the molecule might control the C-terminal domain is supported by the studies showing phosphorylation dependent 14-3-3 binding to N-terminal sequences [13], [39]. 14-3-3 proteins might indirectly influence LRRK2 kinase activity by stabilizing LRRK2 or by affecting its dimer formation [13], [39] which has been shown to influence LRRK2 kinase activity [10]. Although the data presented here do not address how the N-terminal aggregation domain could influence LRRK2 structure, function, stability or binding to targets, the former studies provide a precedent for how sequences far away from either the kinase domain or GTPase might affect these critical functions.

Besides affecting dimerization, LRRK2 mutants that prevent 14-3-3 variants from binding also form aggregates in various cell lines [40]. Exactly how this is accomplished is unknown and at least one other study showing 14-3-3 binding did not observe aggregates [39] making it difficult to interpret these data. However, if the N-terminal sequence affects 14-3-3- binding in some fashion it might also accelerate a process leading to unusual high molecular weight structures or increased complex formation. Which, if any, of these models is relevant will require additional experimentation.

LRRK2 is a very large protein with many protein-protein interaction domains in the N-terminal region. Thus LRRK2 may act as a regulatory scaffolding protein. Since the N-terminus is the region that is most different from the non-PD-associated LRRK1 [14], proteins that are scaffolded by N-terminal LRRK2 might play a role in LRRK2-mediated pathogenesis. Mutations that impair scaffolding might have a dominant negative effect and hence result in a PD phenotype. Finally, many large proteins are also processed. For example, the Huntington's disease protein, huntingtin, is apparently cleaved into many different sized N-termninal fragments by many different proteases including caspases, calpains and a novel endopeptidase [41]. Cleavage may be responsible for releasing the toxic N-terminal repeat containing fragment in this disorder [40]. LRRK2 may be similarly cleaved generating a non-toxic N-terminus and a more-disease prone C-terminus. Although this remains to be tested, huntingtin has established the precedent. Which, if any, of these models is correct for PD awaits further testing.

## Supporting Information

Table S1 Summary of statistical data for figure 2D. Data of Figure 2D were analyzed by one-way ANOVA with Bonferroni *post hoc* test for multiple comparisons.
(DOCX)

Table S2 Summary of statistical data for figure 2E. Data of Figure 2E were analyzed by one-way ANOVA with Bonferroni *post hoc* test for multiple comparisons.
(DOCX)

Table S3 Summary of statistical data for figure 2F. Data of Figure 2F were analyzed by one-way ANOVA with Bonferroni *post hoc* test for multiple comparisons.
(DOCX)

Table S4 Summary of statistical data for figure 3. Data of Figure 3B were analyzed by one-way ANOVA with Bonferroni *post hoc* test for multiple comparisons.
(DOCX)

Table S5 Summary of statistical data for figure 4. Data of Figure 4B were analyzed by one-way ANOVA with Bonferroni *post hoc* test for multiple comparisons.
(DOCX)

Table S6 Summary of statistical data for figure 5. Data of Figure 5 were analyzed by one-way ANOVA with Bonferroni *post hoc* test for multiple comparisons.
(DOCX)

Table S7 Summary of statistical data for figure 6. Data of Figure 6B were analyzed by one-way ANOVA with Bonferroni *post hoc* test for multiple comparisons.
(DOCX)

## References

[1] Greggio E, Bisaglia M, Civiero L, Bubacco L (2011) Leucine-rich repeat kinase 2 and alpha-synuclein: intersecting pathways in the pathogenesis of Parkinson's disease? Mol Neurodegener. 18 6(1): 6. 10.1186/1750-1326-6-6 PMC3035023 21244648

[2] Gasser T (2009) Molecular pathogenesis of Parkinson's disease: insights from genetic studies. Expert Rev Mol Med. 11: e22. 10.1017/S1462399409001148 19631006

[3] Biskup S, Gerlach M, Kupsch A, Reichmann H, Riederer P, et al (2008) Genes associated with Parkinson's syndrome. Neurol. 255: 8–17. 10.1007/s00415-008-5005-2 18787878

[4] Dächsel JC, Farrer MJ (2010) LRRK2 and Parkinson disease. Arch Neurol. 67: 542–7. 10.1001/archneurol.2010.79 20457952

[5] Seol W (2010) Biochemical and molecular features of LRRK2 and its pathophysiological roles in Parkinson's disease. BMB Rep. 43: 233–44. 10.5483/bmbrep.2010.43.4.233 20423607

[6] Wider C, Dickson DW, Wszolek ZK (2010) Leucine-rich repeat kinase 2 gene-associated disease: redefining genotype-phenotype correlation. Neurodegener Dis. 7: 175–9. 10.1159/000289232 PMC2859237 20197701

[7] Wszolek ZK, Pfeiffer RF, Tsuboi Y, Uitti RJ, McComb RD, et al (2004) Autosomal dominant parkinsonism associated with variable synuclein and tau pathology. Neurology 62: 1619–22. 15136696 10.1212/01.wnl.0000125015.06989.db

[8] Daniëls V, Baekelandt V, Taymans JM (2011) On the road to leucine-rich repeat kinase 2 signalling: evidence from cellular and in vivo studies. Neurosignals. 19(1): 1–15. 10.1159/000324488 21430363

[9] Higashi S, Moore DJ, Yamamoto R, Minegishi M, Sato K, et al (2009) Abnormal localization of leucine-rich repeat kinase 2 to the endosomal-lysosomal compartment in lewy body disease. J Neuropathol Exp Neurol. 68: 994–1005. 10.1097/NEN.0b013e3181b44ed8 PMC2768772 19680143

[10] Poulopoulos M, Cortes E, Vonsattel JP, Fahn S, Waters C, et al.. (2011) Clinical and Pathological Characteristics of LRRK2 G2019S Patients with PD. J Mol Neurosci. Dec 23. [Epub ahead of print]. 10.1007/s12031-011-9696-y PMC3335886 22194196

[11] Moore DJ (2008) The biology and pathobiology of LRRK2: implications for Parkinson's disease. Parkinsonism Relat Disord. 14 Suppl 2S92–8. 10.1016/j.parkreldis.2008.04.010 18602856

[12] Greggio E, Lewis PA, van der Brug MP, Ahmad R, Kaganovich A, et al (2007) Mutations in LRRK2/dardarin associated with Parkinson disease are more toxic than equivalent mutations in the homologous kinase LRRK1. J Neurochem. 102: 93–102. 10.1111/j.1471-4159.2007.04523.x 17394548

[13] Nichols RJ, Dzamko N, Morrice NA, Campbell DG, Deak M, et al (2010) 14-3-3 binding to LRRK2 is disrupted by multiple Parkinson's disease-associated mutations and regulates cytoplasmic localization. Biochem J. 430: 393–404. 10.1042/BJ20100483 PMC2932554 20642453

[14] Marín I, van Egmond WN, van Haastert PJ (2008) The Roco protein family: a functional perspective. FASEB J. 22: 3103–10. 10.1096/fj.08-111310 18523161

[15] Mata IF, Wedemeyer WJ, Farrer MJ, Taylor JP, Gallo KA (2006) LRRK2 in Parkinson's disease: protein domains and functional insights. Trends Neurosci. 29: 286–93. 10.1016/j.tins.2006.03.006 16616379

[16] Lu B, Zhai Y, Wu C, Pang X, Xu Z, et al (2010) Expression, purification and preliminary biochemical studies of the N-terminal domain of leucine-rich repeat kinase 2. Biochim Biophys Acta. 1804: 1780–4. 10.1016/j.bbapap.2010.05.004 20493972

[17] Ross OA, Soto-Ortolaza AI, Heckman MG, Aasly JO, Abahuni N, et al. Genetic Epidemiology Of Parkinson's Disease (GEO-PD) Consortium (2011) Association of LRRK2 exonic variants with susceptibility to Parkinson's disease: a case-control study. Lancet Neurol. 10(10): 898–908. 10.1016/S1474-4422(11)70175-2 PMC3208320 21885347

[18] Trovato A, Chiti F, Maritan A, Seno F (2006) Insight into the structure of amyloid fibrils from the analysis of globular proteins. PLoS Comput Biol. 15: e170. 10.1371/journal.pcbi.0020170 PMC1698942 17173479

[19] Trovato A, Seno F, Tosatto SC (2007) The PASTA server for protein aggregation prediction. Protein Eng Des Sel. 20: 521–3. 10.1093/protein/gzm042 17720750

[20] Tartaglia GG, Cavalli A, Pellarin R, Caflisch A (2005) Prediction of aggregation rates and aggregation-prone segments in polypeptide sequences. Protein Sci. 14: 2723–2734. 10.1110/ps.051471205 PMC2253302 16195556

[21] Tartaglia GG, Pawar AP, Campioni S, Dobson CM, Chiti F, et al (2008) Prediction of aggregation-prone regions in structured proteins. J Mol Biol. 380: 425–36. 10.1016/j.jmb.2008.05.013 18514226

[22] West AB, Moore DJ, Biskup S, Bugayenko A, Smith WW, et al (2005) Parkinson's disease-associated mutations in leucine-rich repeat kinase 2 augment kinase activity. Proc Natl Acad Sci 102(46): 16842–7. 16269541 10.1073/pnas.0507360102 PMC1283829

[23] Lotharius J, Dugan LL, O'Malley KL (1999) Distinct mechanisms underlie neurotoxin-mediated cell death in cultured dopaminergic neurons. J Neurosci. 19: 1284–93. 10.1523/JNEUROSCI.19-04-01284.1999 PMC6786015 9952406

[24] Waxman EA, Covy JP, Bukh I, Li X, Dawson TM, et al (2009) Leucine-rich repeat kinase 2 expression leads to aggresome formation that is not associated with alpha-synuclein inclusions. J Neuropathol Exp Neurol. 68: 785–96. 10.1097/NEN.0b013e3181aaf4fd PMC2722758 19535993

[25] Holtz WA, Turetzky JM, Jong YJ, O'Malley KL (2006) Oxidative stress-triggered unfolded protein response is upstream of intrinsic cell death evoked by parkinsonian mimetics. J Neurochem. 99: 54–69. 10.1111/j.1471-4159.2006.04025.x 16987235

[26] Oh Y, Wong S, Moffat M, O'Malley KL (1995) Overexpression of Bcl-2 attenuates MPP+, but not 6-ODHA, induced cell death in a dopaminergic neuronal cell line. Neurobiol. Dis. 2: 157–167. 10.1006/nbdi.1995.0017 9173999

[27] Greggio E, Jain S, Kingsbury A, Bandopadhyay R, Lewis P, et al (2006) Kinase activity is required for the toxic effects of mutant LRRK2/dardarin. Neurobiol Dis. 23: 329–41. 10.1016/j.nbd.2006.04.001 16750377

[28] Caflisch A (2006) Computational models for the prediction of polypeptide aggregation propensity. Curr Opin Chem Biol. 10: 437–44. 10.1016/j.cbpa.2006.07.009 16880001

[29] Tan JM, Wong ES, Lim KL (2009) Protein misfolding and aggregation in Parkinson's disease. Antioxid Redox Signal. 11: 2119–34. 10.1089/ars.2009.2490 19243238

[30] Cuervo AM, Wong ES, Martinez-Vicente M (2010) Protein degradation, aggregation, and misfolding. Mov Disord. 25 Suppl 1S49–54. 10.1002/mds.22718 20187257

[31] Holtz WA, O'Malley KL (2003) Parkinsonian mimetics induce aspects of unfolded protein response in death of dopaminergic neurons. J Biol Chem. 278: 19367–77. 10.1074/jbc.M211821200 12598533

[32] Holtz WA, Turetzky JM, O'Malley KL (2005) Microarray expression profiling identifies early signaling transcripts associated with 6-OHDA-induced dopaminergic cell death. Antioxid Redox Signal. 7: 639–48. 10.1089/ars.2005.7.639 15890008

[33] Arrasate M, Mitra S, Schweitzer ES, Segal MR, Finkbeiner S (2004) Inclusion body formation reduces levels of mutant huntingtin and the risk of neuronal death. Nature 431: 805–810. 15483602 10.1038/nature02998

[34] Ravikumar B, Vacher C, Berger Z, Davies JE, Luo S, et al (2004) Inhibition of mTOR induces autophagy and reduces toxicity of polyglutamine expansions in fly and mouse models of Huntington disease. Nature Genetics 36: 585–595. 15146184 10.1038/ng1362

[35] Ravikumar B, Berger Z, Vacher C, O'Kane CJ, Rubinsztein DC (2006) Rapamycin pre-treatment protects against apoptosis. Hum Mol Genet. 15: 1209–16. 10.1093/hmg/ddl036 16497721

[36] Gomez-Lazaro M, Galindo MF, Concannon CG, Segura MF, Fernandez-Gomez FJ, et al (2008) 6-Hydroxydopamine activates the mitochondrial apoptosis pathway through p38 MAPK-mediated, p53-independent activation of Bax and PUMA. J Neurochem. 104(6): 1599–612. 10.1111/j.1471-4159.2007.05115.x 17996028

[37] Callio J, Oury TD, Chu CT (2005) Manganese superoxide dismutase protects against 6-hydroxydopamine injury in mouse brains. J Biol Chem. 280(18): 18536–42. 10.1074/jbc.M413224200 PMC1885201 15755737

[38] Greggio E, Civiero L, Bisaglia M, Bubacco L (2012) Parkinson's disease and immune system: is the culprit LRRKing in the periphery? J Neuroinflammation. 9 9: 94. 10.1186/1742-2094-9-94 PMC3391996 22594666

[39] Li X, Wang QJ, Pan N, Lee S, Zhao Y, et al (2011) Phosphorylation-dependent 14-3-3 binding to LRRK2 is impaired by common mutations of familial Parkinson's disease. PLoS One. 6(3): e17153. 10.1371/journal.pone.0017153 PMC3046972 21390248

[40] Dzamko N, Deak M, Hentati F, Reith AD, Prescott AR, et al (2010) Inhibition of LRRK2 kinase activity leads to dephosphorylation of Ser(910)/Ser(935), disruption of 14-3-3 binding and altered cytoplasmic localization. Biochem J. 430(3): 405–13. 10.1042/BJ20100784 PMC3631100 20659021

[41] Ross CA, Tabrizi SJ (2011) Huntington's disease: from molecular pathogenesis to clinical treatment. Lancet Neurol. 10: 83–98. 10.1016/S1474-4422(10)70245-3 21163446

