# Supplementary material for: Sequences Located within the N-Terminus of the PD-Linked LRRK2 Lead to Increased Aggregation and Attenuation of 6-Hydroxydopamine-Induced Cell Death
Source: PLoS One. 2012 Sep 13;7(9):e45149. doi: 10.1371/journal.pone.0045149 (PMC3441673; doi:10.1371/journal.pone.0045149)
Supplement: Table S5 — Summary of statistical data for figure 4. Data of Figure 4B were analyzed by one-way ANOVA with Bonferroni post hoc test for multiple comparisons. (DOCX) [file pone.0045149.s005.docx]

**Table S5: Summary of statistical data for figure 4.**


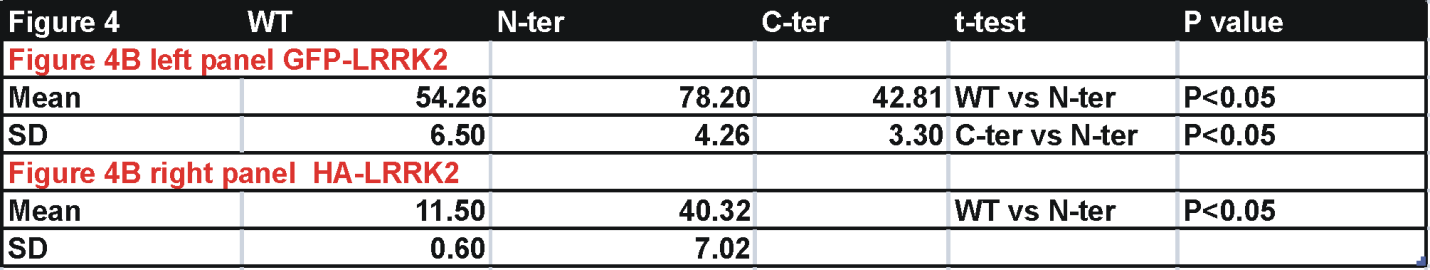


Data of Figure 4B were analyzed by one-way ANOVA with Bonferroni *post hoc* test for multiple comparisons.
